# A case report of recovery of menstrual function following a nutritional intervention in two exercising women with amenorrhea of varying duration

**DOI:** 10.1186/1550-2783-10-34

**Published:** 2013-08-02

**Authors:** Rebecca J Mallinson, Nancy I Williams, Marion P Olmsted, Jennifer L Scheid, Emily S Riddle, Mary Jane De Souza

**Affiliations:** 1 Women’s Health and Exercise Laboratory, 104 Noll Laboratory, Department of Kinesiology, Penn State University, University Park, PA 16802, USA; 2 Toronto General Hospital, University Health Network, Toronto, ON M5G 2C4, Canada; 3 Department of Pediatrics, (current institution for JLS), University at Buffalo, Buffalo, NY 14222, USA

**Keywords:** Amenorrhea, Treatment, Energy intake, Resumption of menses, Bone mineral density

## Abstract

Increasing caloric intake is a promising treatment for exercise-associated amenorrhea, but strategies have not been fully explored. The purpose of this case report was to compare and contrast the responses of two exercising women with amenorrhea of varying duration to an intervention of increased energy intake. Two exercising women with amenorrhea of short (3 months) and long (11 months) duration were chosen to demonstrate the impact of increased caloric intake on recovery of menstrual function and bone health. Repeated measures of dietary intake, eating behavior, body weight, body composition, bone mineral density, resting energy expenditure, exercise volume, serum metabolic hormones and markers of bone turnover, and daily urinary metabolites were obtained. Participant 1 was 19 years old and had a body mass index (BMI) of 20.4 kg/m^2^ at baseline. She increased caloric intake by 276 kcal/day (1,155 kJ/day, 13%), on average, during the intervention, and her body mass increased by 4.2 kg (8%). Participant 2 was 24 years old and had a BMI of 19.7 kg/m^2^. She increased caloric intake by 1,881 kcal/day (7,870 kJ/day, 27%) and increased body mass by 2.8 kg (5%). Resting energy expenditure, triiodothyronine, and leptin increased; whereas, ghrelin decreased in both women. Resumption of menses occurred 23 and 74 days into the intervention for the women with short-term and long-term amenorrhea, respectively. The onset of ovulation and regular cycles corresponded with changes in body weight. Recovery of menses coincided closely with increases in caloric intake, weight gain, and improvements in the metabolic environment; however, the nature of restoration of menstrual function differed between the women with short-term versus long-term amenorrhea.

## Introduction

Exercising women frequently present with a chronic energy deficiency resulting from inadequate caloric intake to compensate for energy expenditure [1,2]. In this population, energy expenditure may be high due to the added energy cost of exercise. Therefore, when daily energy intake does not match energy expenditure, there may be inadequate fuel to support all physiological processes [3]. As a result, the physiological consequences of an energy deficiency involve a cascade of metabolic and hormonal alterations that can suppress the reproductive axis and cause menstrual disturbances such as functional hypothalamic amenorrhea (FHA) and low bone mass [4,5]. The optimal treatment strategy for women with exercise-associated amenorrhea and low bone mass is to target the source of the problem, i.e., the energy deficiency, by initiating a lifestyle intervention that includes an increase in energy intake, and, if necessary, a decrease in exercise energy expenditure (EEE) [6]. Weight gain often occurs secondary to such treatment and has been observed to be a clinically positive outcome associated with resumption of menses and enhanced bone health in exercising women [7-9].

A few investigators have reported case studies of amenorrheic, exercising women who have increased caloric intake and gained weight [7-10]. Dueck et al. [10] and Kopp-Woodroffe et al. [8] described a case study of five amenorrheic athletes who increased caloric intake for 12 to 20 weeks, resulting in weight gain of 1 to 3 kg and the resumption of menses in 3 of 5 participants during the intervention. Fredericson and Kent [7] reported a case study of an amenorrheic athlete who gained weight over the course of 5 years, resulting in the maintenance of normal menstrual cycles and improved bone health. Similarly, Zanker et al. [9] followed an amenorrheic athlete for 12 years and reported increases in bone mineral density (BMD) of the proximal femur with increases in body mass index (BMI). There are, however, no case studies published to date that document the simultaneous changes in energetic and metabolic status and the associated effects on hormonal attributes of reproductive recovery and bone health in amenorrheic exercising women. Indeed, the case studies reported to date have limited their findings solely to the outcome of recovery of menses rather than the documentation of the hormonal aspects of menstrual recovery that include estrogen exposure, progesterone exposure, and ovulation over the course of 12 months of increasing calorie intake. The absence of detailed reports describing the metabolic and hormonal environment surrounding resumption of menses in exercising women with FHA has resulted in a lack of evidence on which to base effective dietary treatment strategies. As such, the value of this case report lies in the opportunity to study the manifestation and resolution of this complex problem using detailed hormonal analyses in an effort to gain a better understanding about the interplay of factors that may contribute to the induction and reversal of FHA in exercising women.

Therefore, the purpose of this case report was to compare and contrast the recovery of two exercising women with current FHA of varying duration (short-term vs. long-term) to a 12-month nutritional intervention. Thus, this case report will describe, in detail, the changes in energetic status, and the hormonal aspects of recovery of menstrual function and bone health in two amenorrheic exercising women.

### Nutritional intervention methods

#### Study design

For the purpose of this case report, two exercising amenorrheic women (aged 19–24 years) with current amenorrhea of short (3 months) and long (11 months) duration were chosen to demonstrate the impact of increased caloric intake on the hormonal aspects of recovery of menstrual function and bone health. The two individuals were chosen because they both demonstrated good compliance to an intervention of 12 months of increased caloric intake targeted to exceed baseline total energy expenditure (TEE) needs by 20-30%, and the ongoing nature of the intervention precludes inclusion of the entire sample of women that participated in the intervention. Both women successfully resumed menses. The presence of amenorrhea at the beginning of the intervention was confirmed by the analysis of daily urinary excretion of estrone-1-glucuronide (E1G) and pregnanediol glucuronide (PdG) metabolites for one 28-day monitoring period. Both women were recreationally active, engaging in > 7 hours of exercise per week at baseline. The primary outcome variables in the 12-month intervention were indices of energy status, bone health and menstrual status.

#### Inclusion criteria

The two women in this case report were exercising women who met the following inclusion criteria: 1) age 18–35 years, 2) BMI 16–25 kg/m^2^[11], 3) weight stable (± 2 kg) for the past 6 months, 4) no history of any serious medical conditions, 5) no current clinical diagnosis of an eating or psychiatric disorder, 6) non-smoking, 7) no medication use that would alter metabolic or reproductive hormone concentrations, 8) ≥ 3 hrs/wk aerobic exercise, 9) no menses for the past 3 months, and 10) no history of a clinical diagnosis of polycystic ovarian syndrome (PCOS), or a free androgen index (FAI), calculated as (total testosterone (nmol/L)/sex hormone binding globulin (SHBG) (nmol/L))*100), > 6 [12]. In addition, the women in this case report presented with current amenorrhea of varying duration, i.e., short-term amenorrhea defined as the cessation of menses for <100 days and long-term amenorrhea defined as the absence of menses for >100 days [13].

#### Screening procedures

Participants signed an informed consent approved by the Institutional Review Board at the University of Toronto or Pennsylvania State University. Height and weight were measured, and participants completed questionnaires to assess medical history, exercise and menstrual history, eating behaviors, and psychological health. A physical exam and blood sample was performed to determine overall health. A semi-structured psychological interview was conducted to ensure that the women were not experiencing major psychiatric disorders, and a registered dietitian assessed eating patterns and food preferences. Dual-energy x-ray absorptiometry (DXA) scans were performed to assess BMD and body composition.

#### Baseline procedures

During a 4-week baseline period, menstrual calendars and daily urine samples for the assessment of menstrual function were collected. Body weight was measured weekly. At week 3 of baseline, energetic markers (leptin, ghrelin, total triiodothyronine (TT3)), markers of bone formation and resorption, body composition, resting energy expenditure (REE), and dietary intake were assessed. Participants also completed a test of aerobic fitness.

#### Classification of baseline menstrual status

Upon study entry, classification of menstrual status was based on self-reported menstrual history, which was confirmed by a 28-day urinary profile of E1G, PdG, and luteinizing hormone (LH) profiles during a 4-week baseline period. FHA was assessed by confirming a negative pregnancy test, normal endocrine panel, no menses in the past 90 days, and documentation of chronically suppressed E1G and PdG profiles observed during the baseline period.

#### Intervention procedures for energy calculations

Both participants were asked to increase their caloric intake 20-30% above baseline TEE while maintaining their usual exercise training regimen. For the purpose of this report, baseline TEE was operationally defined as the sum of REE and purposeful EEE. Energy bars that contained approximately 250–300 kilocalories (1,046-1,255 kJ) were provided by the research staff to increase caloric intake. The target increase in caloric intake was gradually achieved by a slow increase in calories during the first several weeks of the intervention to encourage compliance. A registered dietitian met with the participants regularly to provide strategies to meet the target caloric intake. Participants also regularly met with a clinical psychologist or licensed clinical social worker to monitor general psychological health.

#### Assessment of menstrual function during the intervention

Menstrual function was monitored daily during the intervention by assessing urinary excretion of E1G, PdG, and LH metabolites and the presence of menses as self-reported on monthly calendars. The methods used for the assessment and categorization of menstrual cycles are detailed and have previously been published [2].

#### Recovery of menstrual function categories

To describe the recovery of menstrual function, we classified recovery using several definitions of recovery that ranged in hormonal and clinical relevance. Recovery Category 1 was described simply as “recovery of menses.” The successful recovery of menses after the baseline period was defined as the first occurrence of menstrual bleeding during the intervention. For further analysis of the recovery of menstrual function, Recovery Category 2 was described as resumption of menses preceded by ovulation based on increases in urinary E1G (above 35 ng/ml), PdG (above 2.5 μg/ml), and mid-cycle LH (above 25 mIU/ml) concentrations [2,14]. Recovery Category 3 was described as resumption of menses followed by at least 2 menstrual cycles of less than 36 days each.

#### Anthropometrics

Total body weight was measured by a digital scale during each week of the baseline period and every two weeks during the intervention. Height was measured during the screening period, and BMI was calculated as a ratio of weight to height (kg/m^2^). Baseline values for body weight and BMI were reported as the average of all baseline and screening measurements.

#### Eating behavior assessment

Participants completed the Three Factor Eating Questionnaire (TFEQ) and Eating Disorder Inventory-2 (EDI-2) at screening and at months 2, 3, 6, 9, and 13 (post-study) to assess eating behavior. The TFEQ is a 51-item questionnaire with three subscales – cognitive dietary restraint (CDR), disinhibition, and hunger. Cognitive dietary restraint was evaluated according to the following ranges established by Stunkard and Messick [15]: 0–10 indicated low CDR, 11–13 indicated high CDR, and 14–21 indicated the clinical range. The EDI-2 is a 91-item questionnaire with 8 subscales and 3 provisional subscales, as previously reported [16]. Scores on the first 8 subscales were compared to published means and 95% confidence intervals of eating disorder patients and non-patient college females to assess for symptoms of disordered eating and associated psychological features [17].

#### Body composition and bone mineral density

DXA scans of the total body, lumbar spine, and dual femur were performed to assess body composition and BMD. Body composition was measured at screening and baseline and during months 1, 2, 3, 6, 9, and 13 (post-study). BMD was assessed at all three sites at screening, month 6, and month 13 (post-study). The participants were scanned on either a GE Lunar Prodigy or Lunar iDXA (GE Lunar Corporation, Madison, WI). Consistent with the International Society of Clinical Densitometry guidelines, a cross calibration study was performed to remove systematic bias between the systems as previously published [18].

#### Dietary energy intake

Dietary energy intake was assessed from 3-day diet logs (2 weekdays and 1 weekend-day) completed during week 3 of baseline and each month during the intervention as previously published [18]. Participants met with a registered dietitian regularly who trained them how to record dietary intake accurately and reviewed the completed energy intake logs. Participants received written guidelines regarding proper measurement and reporting of food portions and preparation.

#### Resting energy expenditure

REE was determined by indirect calorimetry during week 3 of baseline and months 2, 3, 6, 9, and 13 (post-study) (Sensormedics Vmax metabolic cart, Yorba Linda, CA). Methods explaining the measurement of REE have been published in detail elsewhere [18]. Predicted REE (pREE) was also calculated using the Harris Benedict equation [19]. We compared the lab-assessed REE to the predicted REE (REE/pREE) to estimate how much the measured REE deviated from the predicted REE. A reduced ratio of measured REE to Harris-Benedict predicted REE of 0.60-0.80 has been reported during periods of low body weight and prior to refeeding in anorexic women [20-22]. We have previously published data using a ratio of REE/pREE <0.90 as the operational definition of an energy deficiency [1,4,16,23]. As such, in this study, a ratio <0.90 was used to discriminate between being energy deficient and energy replete.

#### Purposeful exercise energy expenditure

Purposeful EEE was estimated at baseline and monthly during the intervention using a Polar heart rate monitor. Participants completed exercise logs where all purposeful exercise sessions greater than 10 minutes in duration were recorded for a 7-day period. Energy expended during these purposeful exercise sessions was measured using the OwnCal feature of the Polar S610 or RS400 heart rate monitors (Polar Electro Oy, Kempele, Finland) [24]. The OwnCal feature has been validated for the use in calculating EEE from heart rate. The Polar S601 and RS400 hear rate monitors include rest in their estimation of energy expenditure. To estimate only EEE, we subtracted the most recently measured REE (kcal/min) from the Polar heart rate monitors’ estimation of energy expenditure. For purposeful exercise sessions in which participants did not wear the Polar S610 or RS400 heart rate monitors, the Ainsworth et al. [25,26] compendiums of physical activities were used to determine the appropriate metabolic equivalent (MET) level for the exercise performed [27]. To calculate the energy expended during the exercise session, the MET level was multiplied by the duration (min) of the exercise session and the measured REE (kcal/min). The MET value includes a resting component. To estimate only EEE, we subtracted the most recently measured REE (kcal/min) from this value.

Participants also recorded the type and duration of purposeful physical activity using daily exercise logs to provide a measure of exercise volume during the study.

#### Exercise testing

Maximal aerobic capacity (VO_2max_) was measured during a progressive treadmill test to volitional exhaustion using an on-line MedGraphics Modular VO_2_ System (St Paul, MN) or SensorMedics Vmax metabolic cart (Yorba Linda, Calif., USA) during week 3 of baseline using methods previously published [28].

#### Urinary reproductive hormone measurements

To determine estrogen and progesterone exposure, E1G and PdG urinary metabolites were assessed using a modified trapezoidal integrated area under the curve (AUC) technique. To calculate AUC, the hormone concentrations for two consecutive days of the cycle were averaged; these averages were then summed to provide AUC for the cycle. The methods for measuring urinary reproductive hormones have been previously published [2]. The inter-assay coefficients of variation for high and low internal controls for the E1G assay are 12.2% and 14.0%, respectively. The PdG intra- and inter-assay variability was determined in-house as 13.6% and 18.7%, respectively [2,14]. Urinary LH was determined by coat-a-count immunoradiometric assay (Siemens Healthcare Diagnostics, Deerfield, IL). The sensitivity of the LH assay is 0.15 mIU/ml. The intra- and inter-assay coefficients of variation were 1.6% and 7.1%, respectively.

#### Blood sampling

Blood was collected, processed, and stored after an overnight fast between 0700 and 1000 once during week 3 of baseline and once at the end of baseline using methods previously published in detail [18]. The latter two samples were pooled for all baseline hormone analyses. In addition, blood samples were collected during months 2, 3, 4, 5, 6, 9, 13 (post-study).

#### Serum hormone analysis

The metabolic hormones TT3, leptin, and ghrelin were measured using previously published methods [18,29]. Bone markers including pro-collagen type 1 amino-terminal propeptide (P1NP) and collagen type 1 cross-linked C-telopeptide (CTx) were also measured. P1NP was analyzed by radioimmunoassay (RIA) (Immunodiagnostic Systems, Inc., Scottsdale, AZ). The sensitivity of the assay was 2 μg/L. Intra-assay and inter-assay coefficients of variation were between 6.5-10.2% and 6.0-9.8%, respectively. CTx was analyzed by enzyme-linked immunosorbent assay (ELISA) (Immunodiagnostic Systems, Inc., Scottsdale, AZ). The sensitivity of the assay was 0.02 ng/mL. Intra-assay and inter-assay coefficients of variation for the low control were 3.0 and 10.9%, respectively. All samples from a given participant were analyzed in duplicate.

## Case presentation

### Participant 1: long-term amenorrhea

#### Characteristics at baseline

**Figure 1:**
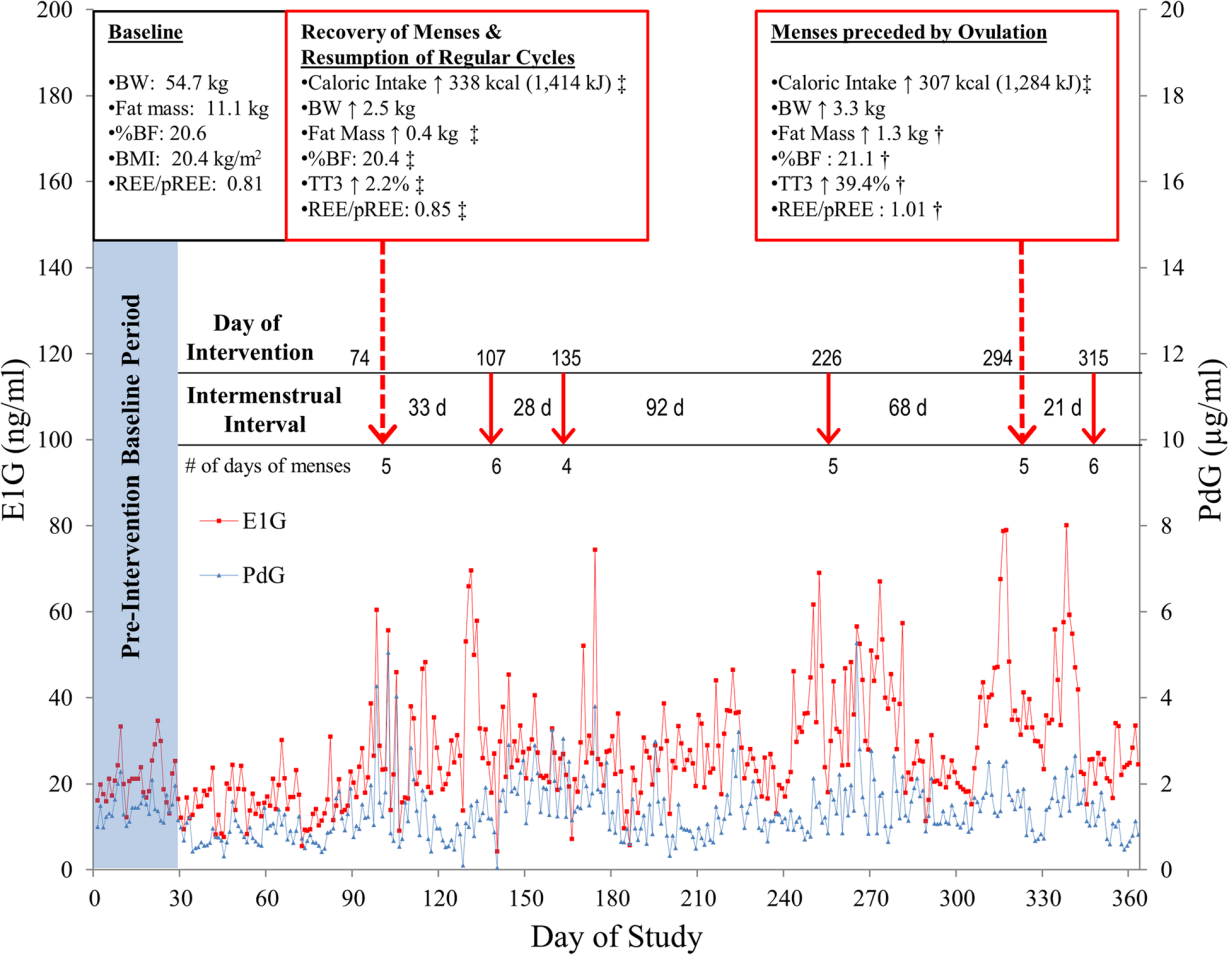
**Reproductive hormone profile for Participant 1.** This figure displays the reproductive hormone profile during the study for Participant 1 and the changes in caloric intake, body weight, and energy status that coincided with each category of menstrual recovery. Arrows indicate menses. Body weight was measured within 1 week of menses. ‡ Indicates data were collected 2 weeks before menses. † Indicates data were collected 6 weeks after menses. %BF: percent body fat; BMI: body mass index; BW: body weight; E1G: estrone-1-glucuronide; PdG: pregnanediol glucuronide; REE/pREE: measured resting energy expenditure/predicted resting energy expenditure; TT3: total triiodothyronine.

This participant was a 19-year old recreationally active college student who participated in a wide variety of activities such as running, weightlifting, rock climbing, hiking, and downhill skiing. At baseline, she reported 12 hours of physical activity each week and averaged about 9 hr/wk of purposeful EEE during the study. Despite presenting with a normal BMI of 20.4 kg/m^2^ and body fat of 20.6%, she had been amenorrheic for 11 months when the intervention commenced and urinary analysis of E1G and PdG confirmed suppressed ovarian activity (Table 1, Figure 1). She presented with a dietary CDR score of 12 which is elevated but not above the clinical threshold of 14 [15]. Scores on the subscales of the EDI-2 were within or below the normal range for college-aged women and did not indicate disordered eating (Table 2). The baseline semi-structured psychological interview revealed that the participant felt good about herself and her healthy eating pattern. There was no evidence of current or past eating disorders. Over the course of the study, Participant 1 reported having no difficulty following the energy intake prescriptions.

**Table 1:** Baseline descriptives of the women

	**Participant 1**	**Participant 2**
**Demographic characteristics**		
Age (yr)	19	24
Height (cm)	164.0	165.5
Weight (kg)	54.7	54.0
BMI (kg/m^2^)	20.4	19.7
Body fat (%)	20.6	22.7
**Reproductive characteristics**		
Age of Menarche (yr)	15	13
Gynecological age (yr)	4	9
Duration of amenorrhea (days)	330	90
Duration until resumption	74	23
(days in intervention)		
# Cycles during intervention	6	9
**Training characteristics**		
Physical activity (min/wk)^*^	761	438
VO_2_max (ml/kg/min)	50.1	43.5

^*^Self-reported exercise during baseline.

BMI: body mass index; VO_2_max: maximal oxygen consumption.

**Table 2:** Baseline, month 6, and post-intervention scores on subscales of the Three Factor Eating Questionnaire and Eating Disorder Inventory-2

**Eating behavior scores**	**Participant 1**	**Participant 2**
Cognitive dietary restraint		
*Baseline*	12	12
*Month 6*	11	12
*Post-Intervention*	12	8
Drive for thinness		
*Baseline*	0	6
*Month 6*	2	3
*Post-Intervention*	1	4
Body dissatisfaction		
*Baseline*	0	6
*Month 6*	0	0
*Post-Intervention*	0	0
Perfectionism		
*Baseline*	7	17
*Month 6*	5	13
*Post-Intervention*	6	12

#### Changes in energetic status

The participant was instructed to gradually increase her daily dietary intake by 500 kcal/day (2,092 kJ/day) which represented an increase of 31% above her baseline energy requirement (TEE) and a target caloric intake for the intervention of 2,600 kcal/day (10,878 kJ/day). The participant’s caloric intake was 2,143 kcal/day (8,966 kJ/day) at baseline and increased to an average intake of 2,419 kcal/day (10,121 kJ/day) during the intervention. Exercise volume remained relatively constant throughout the intervention, ranging from 7 to 12 hr/wk. Weekly EEE averaged 685 kcal/day (2,866 kJ/day) with a range of 319 to 1,013 kcal/day (1, 335 – 4,238 kJ/day).

During the intervention, the participant demonstrated a progressive weight gain of 1.8 kg at month 3, 2.1 kg at month 6, and 4.2 kg after month 12 of the intervention when compared to her baseline weight. The increase in weight coincided with an increase in BMI from 20.4 kg/m^2^ at baseline to 22.0 kg/m^2^ after month 12. Fat and lean mass (LBM) increased by 11.7% and 8.3%, respectively, which translated to an increase of 1.3 kg of fat mass and 3.4 kg of lean mass. Percent body fat increased from 20.6% to 21.1%. The greatest increase in fat mass was observed at month 9 with an increase of 2.0 kg from baseline, and a concomitant increase in circulating leptin concentration of 105.7% from baseline to month 9. An increase in REE from 27.20 to 32.61 kcal/day/kg LBM (113.8 to 136.4 kJ/day/kg LBM) was observed from baseline to month 12. The REE/pREE ratio also increased from 0.81 at baseline to 1.01 at the end of the study, demonstrating an improvement in energy status. Further evidence of an improved energy state is corroborated by a 39.4% increase in TT3 and a 59.2% decrease in ghrelin concentrations (Table 3).

**Table 3:** Baseline measurements and the 6-month and 12-month percent change for metabolic hormone concentrations

	**Participant 1**	**Participant 2**
**Metabolic hormones**		
Leptin (μg/ml)	5.1	2.4
*6 month % change*	−19.8	230.9
*12 month % change*	−17.3	279.8
Total Ghrelin (pmol/L)	534.8	490.3
*6 month % change*	−35.9	−15.2
*12 month % change*	−59.2	−12.1
Total Triiodothyronine (nmol/L)	0.82	1.06
*6 month % change*	8.0	6.3
*12 month % change*	39.4	31.5

Ghrelin conversion: pg/ml x 0.296 = pmol/L.

Triiodothyronine conversion: ng/dl x 0.0154 = nmol/L.

#### Changes in menstrual status

After 2.5 months (74 days) in the intervention, menses resumed (Figure 1). However, due to the anovulatory nature of the cycle preceding resumption, estrogen exposure, as assessed by E1G AUC, was not improved from the baseline period to the time period preceding resumption. For the first two months after resumption, two consistently eumenorrheic but anovulatory cycles of 28 to 33 days in length were observed (Figure 1). About 6 months into the intervention, however, she experienced another brief episode of amenorrhea with 92 days elapsing between menses. About 8 months in the intervention and 3 months after her last menses (92 days), she resumed menses for a second time. A long intermenstrual interval of 68 days characterized the first cycle after resumption. During this time, a decrease in caloric intake of approximately 400 kcal/day (1,674 kJ/day) in the face of a consistent volume of EEE was observed. The participant was informed of the decrease in caloric intake and was instructed again to increase her daily energy intake to 2,600 kcal/day (10,878 kJ/day). She was moderately successful, increasing her intake to approximately 2,350 kcal/day (9,832 kJ/day). Consequently, the cycle following the second resumption was ovulatory but characteristic of an inadequate luteal phase, representing the first ovulatory cycle that this participant experienced during the intervention. Estrogen exposure during the 28 days preceding the ovulation-associated menses increased 64.3% compared to the baseline cycle. Furthermore, despite its anovulatory nature, the length of the subsequent and final cycle during the study declined sharply with an intermenstrual interval of 21 days.

#### Changes in bone health

As Table 4 demonstrates, the participant had a low BMD at the lumbar spine at baseline. After the 12-month intervention, no increases in BMD were observed at any skeletal site; however, P1NP, a marker of bone formation, increased by 49.6%.

**Table 4:** Baseline measurements and the 6-month and 12-month percent change for bone marker concentrations and BMD

	**Participant 1**	**Participant 2**
**Bone markers**		
P1NP (μg/L)	52.90	36.95
*6 month % change*	5.6	22.6
*12 month % change*	49.6	51.6
CTx (ng/ml)	0.65	0.64
*6 month % change*	−23.1	−29.0
*12 month % change*	17.7	−36.1
**Bone mineral density**		
Lumbar spine Z-score	−1.6	−1.4
Lumbar spine BMD (g/cm^2^)	0.983	1.056
*6 month % change*	1.7	2.6
*12 month % change*	0.8	2.0
Femoral neck Z-score	0.5^*^	−0.6
Femoral neck BMD (g/cm^2^)	1.062	0.994
*6 month % change*	−2.8	−0.3
*12 month % change*	−4.3	1.4
Hip Z-score	0.0^*^	−1.1
Hip BMD (g/cm^2^)	0.996	0.955
*6 month % change*	−1.3	−0.4
*12 month % change*	−2.0	1.9

^*^Z-score at month 6.

BMD: bone mineral density; CTx: collagen type 1 cross-linked C-telopeptide;

P1NP: pro-collagen type 1 amino-terminal propeptide.

### Participant 2: short-term amenorrhea

#### Characteristics at baseline

This participant was a 24-year old graduate student who participated in approximately 7 hours of exercise each week, consisting of dancing, running, and weight training. She presented with a normal BMI of 19.7 kg/m^2^ and percent body fat of 22.7%; however, at the start of the intervention, she had not had menses for three months, and her menstrual history revealed multiple extended episodes of amenorrhea (Table 1). Menarche occurred at 13 years of age. At age 16, she experienced an 8-month episode of amenorrhea. After she resumed menses, she had regular cycles until the age of 21 years when she experienced a prolonged episode of amenorrhea for 2.5 years that she associated with low food intake, stress, and excessive exercise. During this time of amenorrhea, she weighed 43 kg but gained about 10 kg to bring her to the weight of 53.8 kg which was measured at the baseline period of this report. Ten months prior to starting this study, she resumed sporadic menses, reporting 5 cycles during those months. Menstrual disturbances were still present, however, as confirmed by self-reported long cycles and suppressed concentrations of E1G and PdG measured at baseline.

The participant presented with an elevated but not clinical dietary cognitive restraint score of 12 and scores that were above normal for college-aged women and within the range for eating disorder patients for the following four subscales of the EDI-2: ineffectiveness, perfectionism, interpersonal distrust, and interoceptive awareness [17] (Table 2). The baseline semi-structured psychological interview revealed that Participant 2 had a history of clinical diagnosis of anorexia nervosa and although she no longer met criteria for a clinical eating disorder, she continued to have associated characteristics such as perfectionism, social anxiety and reservations about trusting others.

#### Changes in energy status

The participant was instructed to gradually increase daily dietary intake by 400 kcal/day (1,674 kJ/day), representing an increase of 27% above her baseline energy requirements (TEE) and a target caloric intake of 1,900 kcal/day (7,950 kJ/day). Her caloric intake increased from 1,482 kcal/day (6,201 kJ/day) at baseline to an average intake of 1,917 kcal/day (8,021 kJ/day) for the first six months of the study. During the latter 6 months, an average intake of 1,838 kcal/day (7,690 kJ/day) was observed. Exercise volume ranged from 3 to 7 hr/wk during the intervention with the exception of one month during which 10 hours of purposeful EEE were reported. Weekly EEE averaged 237 kcal/day (992 kJ/day) with a range of 30 to 508 kcal/day (126–2,125 kJ/day).

The participant gradually gained weight for the first 6 months of the intervention such that by month 6, her weight had increased by 2.4 kg. After 12 months, the total weight gain was 2.8 kg, indicating that her weight remained relatively stable during the last 6 months of the study. Coinciding with this increase in weight, BMI increased from 19.7 kg/m^2^ to 20.7 kg/m^2^, and fat mass steadily increased with a total gain of 2.2 kg (17.5% increase). Interestingly, lean mass decreased 1.4 kg (−3.3%) after 12 months which primarily occurred during the last 6 months of the study. Leptin concentrations increased during the study (279.8% increase) (Table 3). Improvement in energy status was demonstrated by an increase in REE from 28.1 kcal/day/kg LBM (117.6 kJ/day/kg LBM) to 32.8 kcal/day/kg LBM (137.2 kJ/day/kg LBM) at the completion of the study which coincided with an increase in the REE/pREE ratio from 0.87 to 0.94. Further evidence for this improved energy state was an increase in TT3 (31.2%) and a decrease in ghrelin (−12.1%) (Table 3).

#### Changes in menstrual status

**Figure 2:**
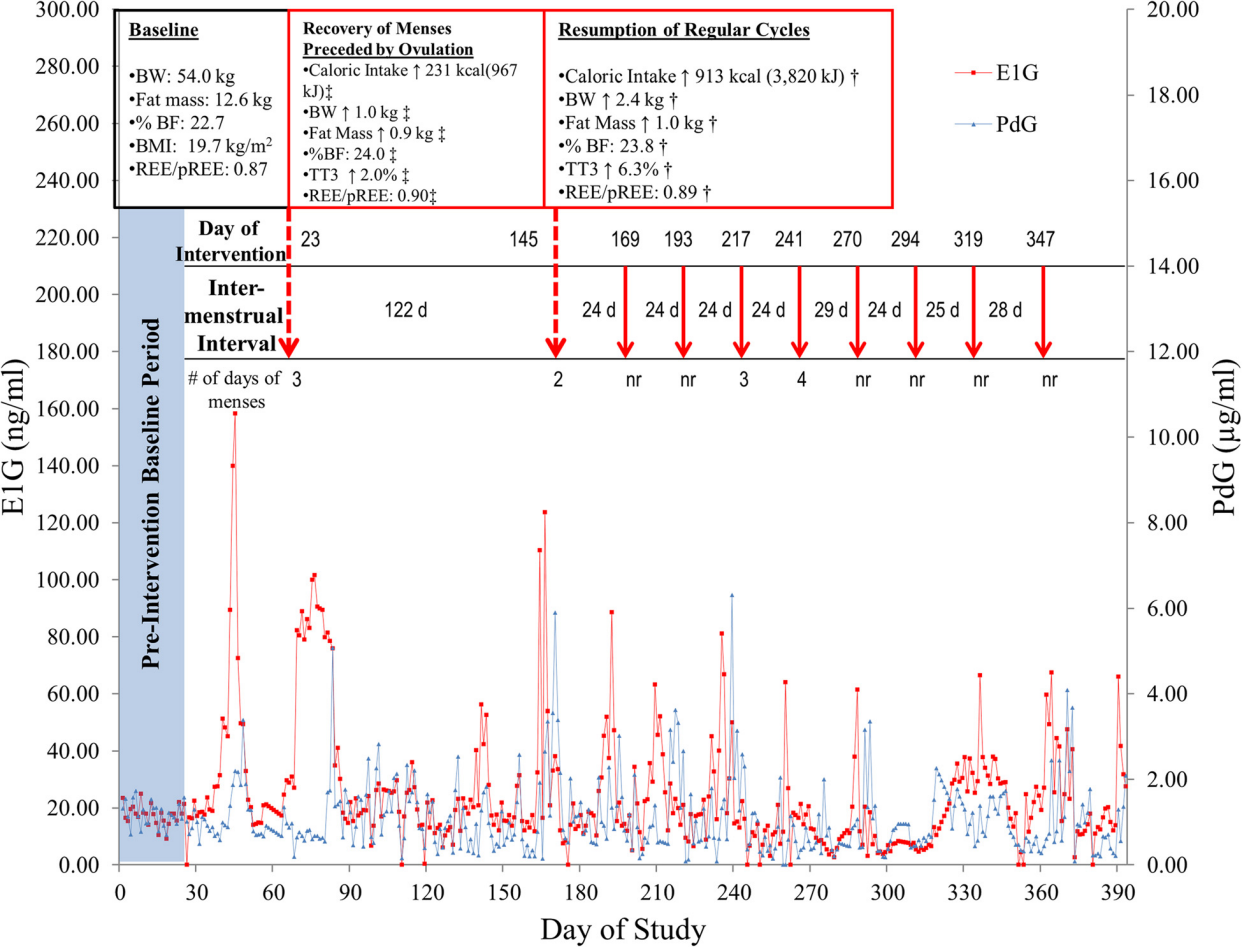
**Reproductive hormone profile for Participant 2.** This figure displays the reproductive hormone profile during the study for Participant 2 and the changes in caloric intake, body weight, and energy status that coincided with each category of menstrual recovery. Arrows indicate menses. ‡ Indicates data were collected 5 weeks after menses. † Indicates data were collected 3 days after menses. %BF: percent body fat; BMI: body mass index; BW: body weight; E1G: estrone-1-glucuronide; nr: not reported; PdG: pregnanediol glucuronide; REE/pREE: measured resting energy expenditure/predicted resting energy expenditure; TT3: total triiodothyronine.

The participant resumed menses 23 days after the start of the intervention, an event that was preceded by ovulation (Figure 2). Estrogen exposure increased 139.4% from baseline to the cycle preceding the resumption of menses. However, menses was not reported for the following 4 months and chronically suppressed concentrations of E1G and PdG were observed, confirming the presence of another episode of amenorrhea. During this period of amenorrhea, body weight and caloric intake decreased slightly toward baseline values then increased again, leading to a second resumption of menses 144 days (~5 months) into the intervention. For the remaining 7 months of the study, 8 more cycles were reported, with consistent cycle lengths of 24 to 29 days (Figure 2). Despite consistent intermenstrual intervals, the cycles were characterized by subtle menstrual disturbances. Of the 10 cycles reported during the study, 6 were ovulatory and 4 were anovulatory. Of the ovulatory cycles, all of them displayed a luteal phase defect. Four cycles were characterized by both a short and inadequate luteal phase, one cycle had just a short luteal phase, and one cycle had an inadequate luteal phase.

#### Changes in bone health

As depicted in Table 4, low BMD at the lumbar spine and hip were observed at baseline. No significant increases in BMD were observed; however, P1NP increased by 51.6% and CTx decreased 36.1%, demonstrating a favorable change in bone turnover.

## Discussion

This case report examined the effects of a 12-month controlled intervention of increased caloric intake in two exercising women with current amenorrhea of varying duration and documents for the first time the simultaneous response of markers of energetic status, daily changes in reproductive hormones, and markers of bone health. The two women in this case report successfully gained weight and resumed menses in response to the non-pharmacological intervention of increased caloric intake. We also document the onset of ovulatory function and regular inter-menstrual intervals in these women and highlight the improved energetic milieu that preceded the reproductive milestones.

Resumption of menses successfully occurred in both women with an intervention that increased caloric intake rather than decreased EEE, a strategy that may be attractive to both athletes and coaches because it does not interfere with training volume or intensity. The increase in caloric intake was tailored to the individualized energy requirements of each participant and was associated with an increase in body weight and an improved energy status. On average, an increase in body weight of 3.5 kg was observed commensurate with an increase in REE from baseline to the completion of the study. In our lab, we have used the ratio of REE/pREE as an indicator of energy status and have operationally defined an energy deficiency as a ratio <0.90 [4,16,23]. Both women presented with a ratio <0.90 at baseline, indicative of an energy deficient state. Previous reports of the REE/pREE ratio in amenorrheic exercising women have ranged from 0.80 to 0.95 [4,28,30] and in anorexic women from 0.60 to 0.80 [20-22]. The two women in this case report resumed menses and experienced increases in REE such that the REE/pREE ratio improved to above 0.90 at the completion of the intervention, indicative of an improvement in energy status and reversal of the energy deficiency.

Likewise, changes in TT3 and ghrelin concentrations paralleled the changes in body weight and REE and provide support for the critical importance of an energy replete state for the successful resumption of menses. Interestingly, fasting concentrations of TT3 increased and ghrelin decreased during the intervention in both women. TT3 is a well-known marker of energy status and is often suppressed among amenorrheic athletes when compared to their ovulating counterparts and sedentary women [1,28]. In fact, it has been shown in the non-human primate model that induction of amenorrhea via an increase in exercise volume and caloric expenditure results in a significant decrease in circulating concentrations of TT3 that is reversed with increases in caloric intake and resumption of menses [31]. Ghrelin, on the other hand, is an orexigenic hormone that regulates appetite and is commonly elevated among amenorrheic exercising women [28,32]. Therefore, an increase in fasting concentrations of TT3 and a decrease in ghrelin provide evidence for improvements in energy status.

In response to the intervention, each woman successfully resumed menses as defined by the occurrence of menstrual bleeding and experienced at least one cycle that was preceded by ovulation. However, in association with varying duration of amenorrhea, the changes observed for each woman in dietary intake, body weight, and the energetic environment that were associated with the reproductive milestones varied. For Participant 1 with long-term amenorrhea, it appeared that weight gain greater than 2 kg coincided with recovery of menses and a gain of about 3 kg coincided with ovulation. However, for Participant 2 with short-term amenorrhea, minimal change in weight prior to the first menses during the study was observed, but approximately 2 kg of weight gain was necessary before the onset of regular cycles. It should be noted, however, that upon entrance into the study Participant 2 reported experiencing long intermenstrual intervals in the previous year, indicative of an oligomenorrheic profile. Thus, it appears that upon commencement of the intervention, this woman was presumably in the early stages of recovery, and the first menses observed during the study may have been another sporadic menses similar to those that she had been experiencing for the past 10 months. Robust increases in caloric intake and subsequent weight gain may have aided resumption of regular intermenstrual intervals as evidenced by consistent cycles of 24 to 29 days in length for the last 7 months of the study.

Body composition and the metabolic milieu at baseline may have played a role in both the time to and quality of recovery of menses. At baseline, both women presented with a BMI and percent body fat within the normal range for exercising women; however, Participant 2 (short-term amenorrhea) presented with a greater percent body fat at baseline than Participant 1. Body fat has been recognized as playing an important permissive role in reproductive function through the effects of leptin, an adipocyte-derived metabolic hormone [33,34]. Leptin binds to receptors in the hypothalamus, stimulating the release of gonadotropin-releasing hormone [35,36] and thereby playing a regulatory role in reproductive function via its influence on gonadotropin pulsatility and reproductive steroid production [37]. Alterations in leptin secretion parallel changes in fat mass; however, leptin secretion is also sensitive to acute alterations in circulating concentrations of glucose [38] and insulin [39]. Consequently, a change in leptin concentration may occur prior to a change in fat mass [37]. In this way, leptin may be mediating recovery of menstrual function prior to notable changes in fat mass. In this case report, Participant 2 with short-term amenorrhea demonstrated robust increases in fat mass and leptin concentration within the first 6 months of the intervention and, coinciding with this increase in leptin, displayed both an ovulatory cycle and resumption of regular cycles early in the intervention. On the other hand, Participant 1 with long-term amenorrhea gained minimal fat mass and showed no increase in leptin concentration during the first 6 months of the intervention despite an increase in circulating TT3. Interestingly, she did not experience an ovulatory cycle until month 11 after demonstrating a gain in fat mass of 2.0 kg and increase in leptin concentration of 106% at month 9 of the intervention. Of further interest is that body fat and leptin concentration decreased again by month 12; whereas, REE and TT3 concentration continued to increase during the last few months of the intervention. Therefore, the woman with short-term amenorrhea seemed to recover faster secondary to robust increases in fat mass and leptin early in the intervention; whereas, the woman with long-term amenorrhea required more time to achieve an ovulatory cycle and demonstrated cycles of greater inconsistency, coinciding with inconsistent changes in fat mass and circulating leptin concentration. As such, in agreement with other investigators [34], leptin concentration, which is strongly correlated with percent body fat [18], likely plays a role in recovery of menses. Furthermore, the woman with long-term amenorrhea (Participant 1) maintained a lower percent body fat as well as greater exercise volume throughout the intervention compared to the woman with short-term amenorrhea (Participant 2), providing further potential reasons for the differences observed during recovery of menstrual function.

Of interest, however, is that neither woman experienced complete recovery of menstrual function as defined by the occurrence of consistent ovulation and regular cycles of 26 to 35 days during the course of the intervention. Despite the onset of menses, subtle menstrual disturbances or long intermenstrual intervals were observed throughout the study. The presence of subtle menstrual disturbances in exercising women who are regularly cycling is not uncommon [2,14]. In fact, it has been reported that about 52% of exercising women experience subtle menstrual disturbances in the face of apparently regular cycles [2]. Thus, it is plausible that women who are recovering from amenorrhea may also experience these subtle menstrual disturbances prior to complete recovery of optimal menstrual function which may require more time than 12 months.

Furthermore, it is notable that both women experienced a decrease in energy intake during the intervention that corresponded with long intermenstrual intervals consistent with the definition of amenorrhea and oligomenorrhea. This non-compliance with the prescribed energy intake, whether inadvertent or intentional, for a period of time during the intervention may have also contributed to the time course of recovery of menstrual function and the lack of complete recovery of optimal menstrual function. However, both women increased caloric intake again after this period of non-compliance, coinciding with ovulation and the onset of regular cycles for Participant 1 and 2, respectively. These events further demonstrate the importance of adequate energy intake on menstrual function among exercising women.

No improvements in bone health for either woman were observed, likely secondary to the relatively short intervention of 12 months. For bone health outcomes, a longer intervention of 18 to 24 months may be required to realize significant changes in bone density and strength. Neither woman demonstrated a clinically significant increase in BMD as defined by a change that exceeded the least significant change; however, P1NP, a marker of bone formation, increased by approximately 50% in both women. This favorable change in bone turnover may indicate that more significant BMD changes may have been observed if the participants were followed for a longer duration of time.

Other case studies of amenorrheic athletes who gained weight demonstrated significant improvements in bone health [7,9]. Frederickson et al. [7] reported a 25.5% and 19.5% increase in lumbar spine and hip BMD, respectively, over 8 years after a gradual weight gain of 17 kg in an athlete who presented with amenorrhea and very low BMD. Similarly, Zanker et al. [9] observed a 16.9% increase in hip BMD after weight gain of 8 kg over 36 months in an endurance athlete with primary amenorrhea and low BMD. These case studies demonstrate that weight gain can lead to significant increases in BMD if an adequate energy state is achieved and adequate time has passed to allow for measurable changes in BMD. It must be noted, however, that in larger samples which have primarily been composed of anorexic women and adolescents, investigators have reported both minimal changes and increases in BMD with weight gain [40,41], highlighting the need for more research in this area.

Strengths of this case report include the detailed assessments of energy status, the metabolic environment, menstrual function, and bone health for a 12-month period. Furthermore, characterizing changes and improvements in menstrual function using urinary metabolites of reproductive hormones collected daily for 12 months provides the opportunity to examine subtle changes in menstrual function that coincide with improvements in the energetic and metabolic environments. A limitation of this case report is the omission of non-exercise activity thermogenesis from the calculation of TEE as a result of problems encountered with the accelerometers used for the study, therefore resulting in a lack of reliable data for this variable.

## Conclusion

This case report provides further support for the role of energy deficiency in menstrual dysfunction among exercising women and the benefits of an adequate energy intake on reproductive health. Resumption of menses coincided closely with weight gain and improvements in energy status that were achieved by increases in caloric intake. This case report also demonstrates that the nature of recovery of menstrual function among exercising women with FHA may differ according to individual differences in duration of amenorrhea, body composition, exercise volume, and the metabolic milieu. Therefore, the response to an increase in caloric intake as well as the time course of menstrual recovery is unique to each woman; however, it appears that improvements in energy status are closely linked to improvements in menstrual function. Further research is needed in larger samples to determine the primary contributors to resumption of menses in amenorrheic, exercising women.

### Consent

The participants signed a consent approved by the Institutional Review Board of the Pennsylvania State University (Participant 1) or the University of Toronto (Participant 2) which informed the participants that the data would be published in medical journals without personally identifiable information. A copy of the signed informed consent is available for review upon request.

## Abbreviations

AUC: Area under the curve; BMD: Bone mineral density; BMI: Body mass index; CDR: Cognitive dietary restraint; CTx: Collagen type 1 cross-linked c-telopeptide; DXA: Dual-energy x-ray absorptiometry; E1G: Estrone-1-glucuronide; EDI-2: Eating disorder inventory-2; EEE: Exercise energy expenditure; ELISA: Enzyme-linked immunosorbent assay; FAI: Free androgen index; FHA: Functional hypothalamic amenorrhea; LBM: Lean body mass; LH: Luteinizing hormone; P1NP: Pro-collagen type 1 amino-terminal propeptide; PdG: Pregnanediol glucuronide; PCOS: Polycystic ovarian syndrome; pREE: Predicted resting energy expenditure; REE: Resting energy expenditure; RIA: Radioimmunoassay; SHBG: Sex hormone binding globulin; TEE: Total energy expenditure; TT3: Total triiodothyronine; TFEQ: Three factor eating questionnaire; VO2max: Maximal aerobic capacity.

## Competing interests

The authors declare that they have no competing interests.

## Authors’ contributions

RJM was responsible for data collection, data analysis and interpretation, and the writing of the manuscript. MPO helped with data collection and contributed to the writing of the manuscript. JLS helped with data collection and writing of the manuscript, and ESR participated in data collection, data analysis, and the writing of the manuscript. MJD and NIW designed the study and supervised the data collection, analysis, and interpretation. MJD also supervised the writing of the manuscript. All authors read and approved the final manuscript.
